# Supplementary material for: Structural basis of regulated N-glycosylation at the secretory translocon
Source: Nature. 2025 Nov 19;649(8097):777–84. doi: 10.1038/s41586-025-09756-8 (PMC12804085; doi:10.1038/s41586-025-09756-8)

nature portfolio

https://doi.org/10.1038/s41586-025-09756-8

Supplementary information

Structural basis of regulated N-glycosylation at the secretory translocon

In the format provided by the authors and unedited

Supplementary Figure 1.

Fig. 3e

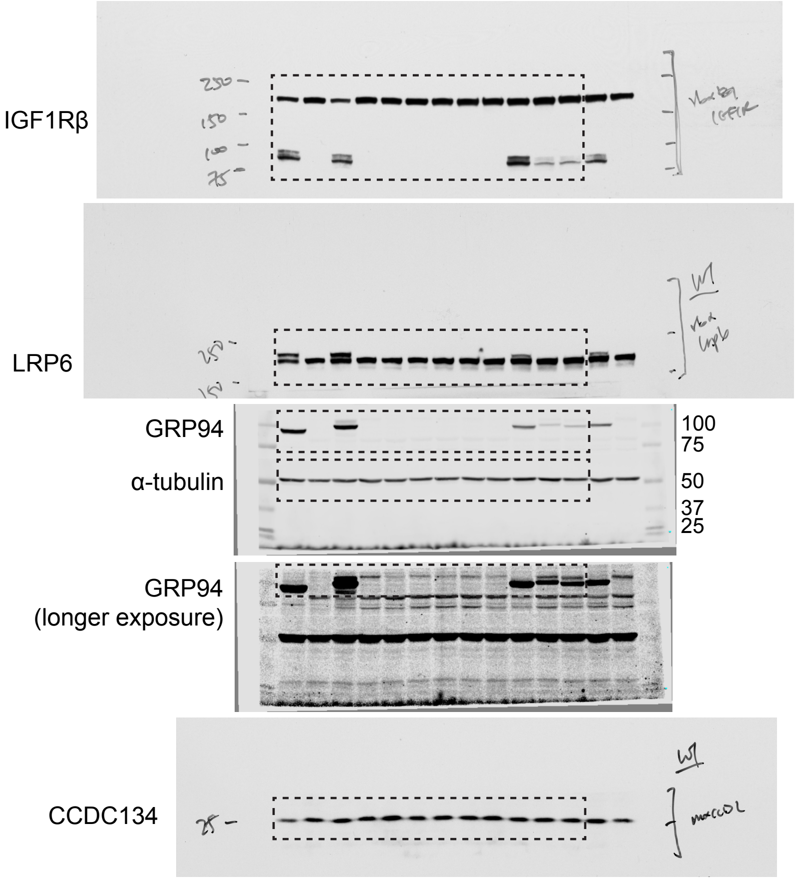

Fig. 3h

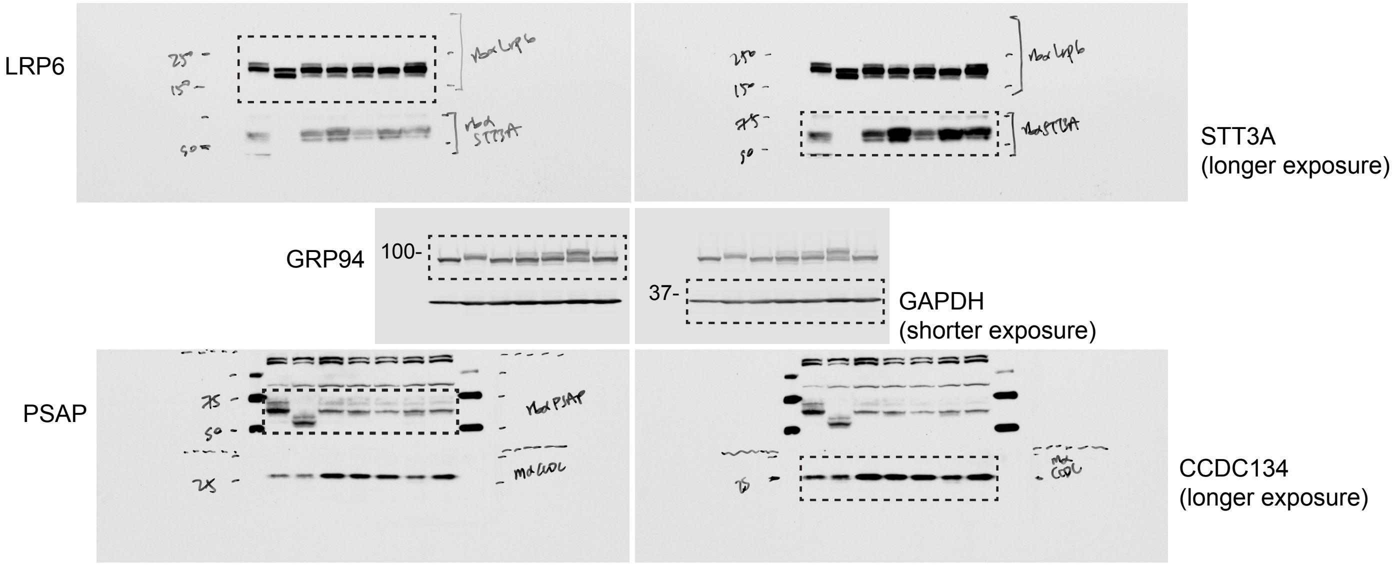

Fig. 4e

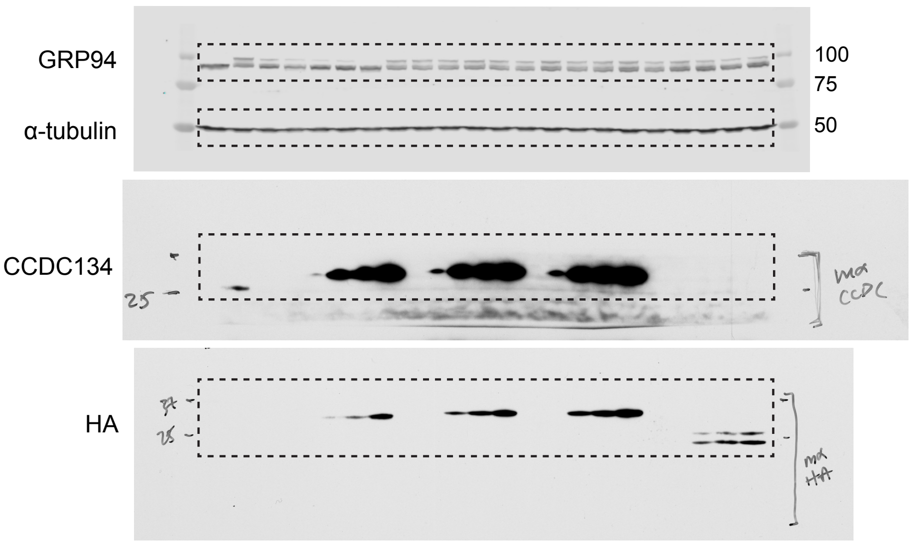
GRP94
100
75
α-tubulin
50
CCDC134
HA

Fig. 4f

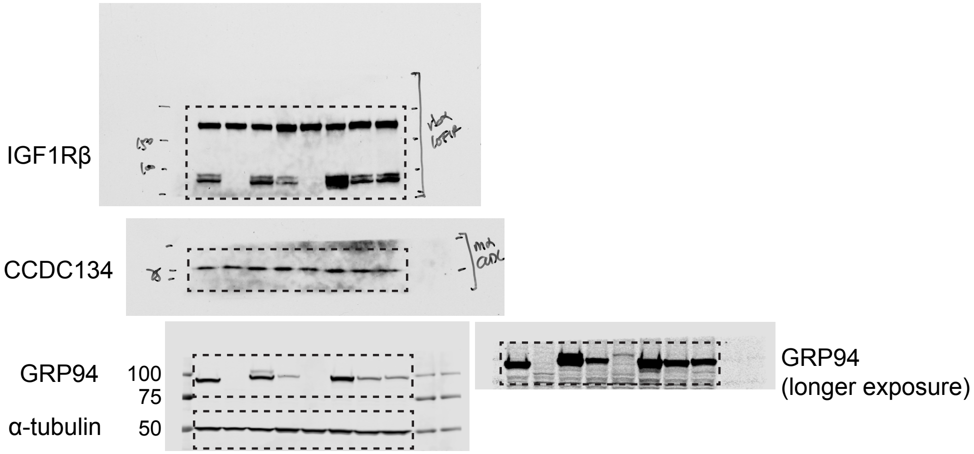
IGF1Rβ
CCDC134
GRP94
100
75
α-tubulin
50
GRP94
(longer exposure)

Extended Data Fig. 1b: Ribosome-bound fraction

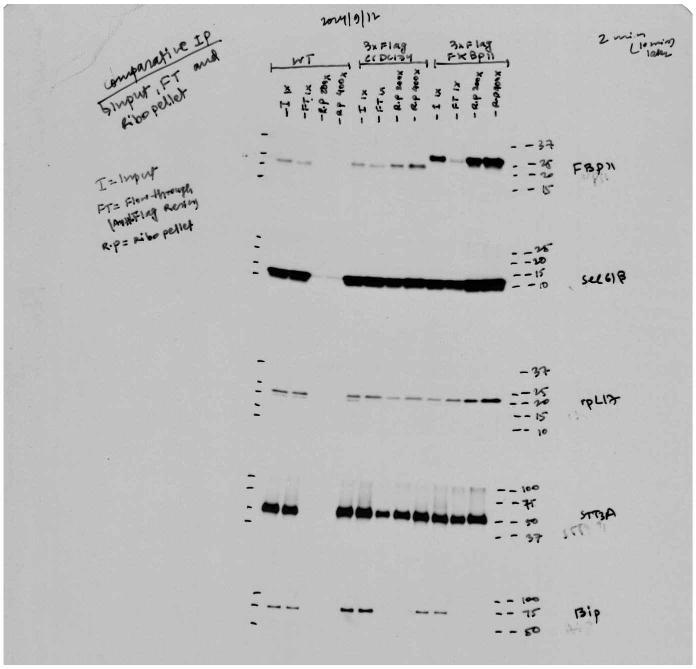

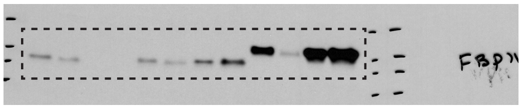

FKBP11

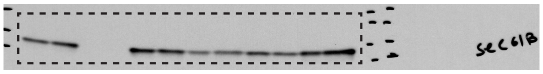

Sec61β

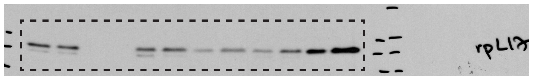

uL22 (RPL17)

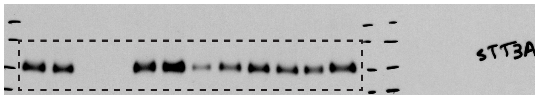

STT3A

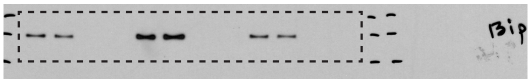

BiP

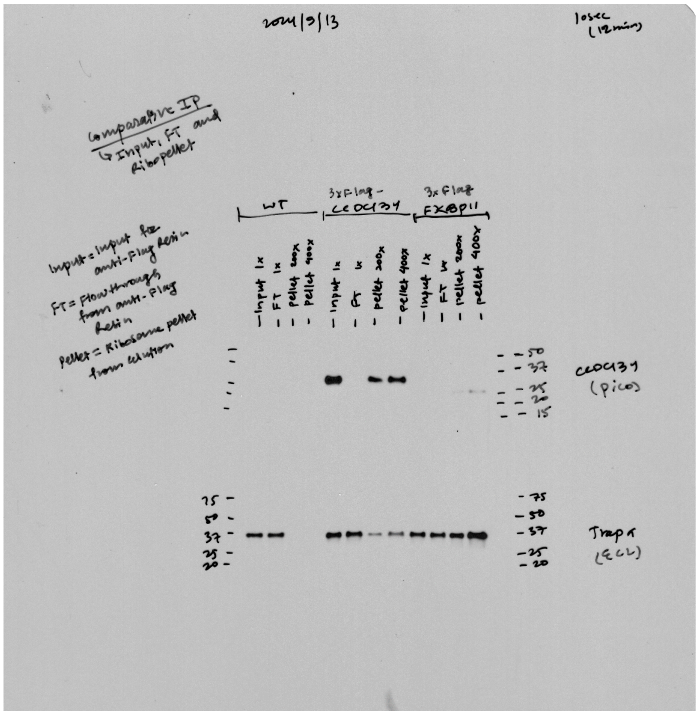

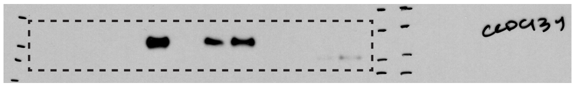

CCDC134

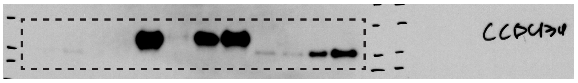

CCDC134 (longer exposure)

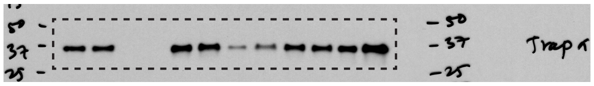

TRAPα

Extended Data Fig. 1b: Ribosome-free fraction

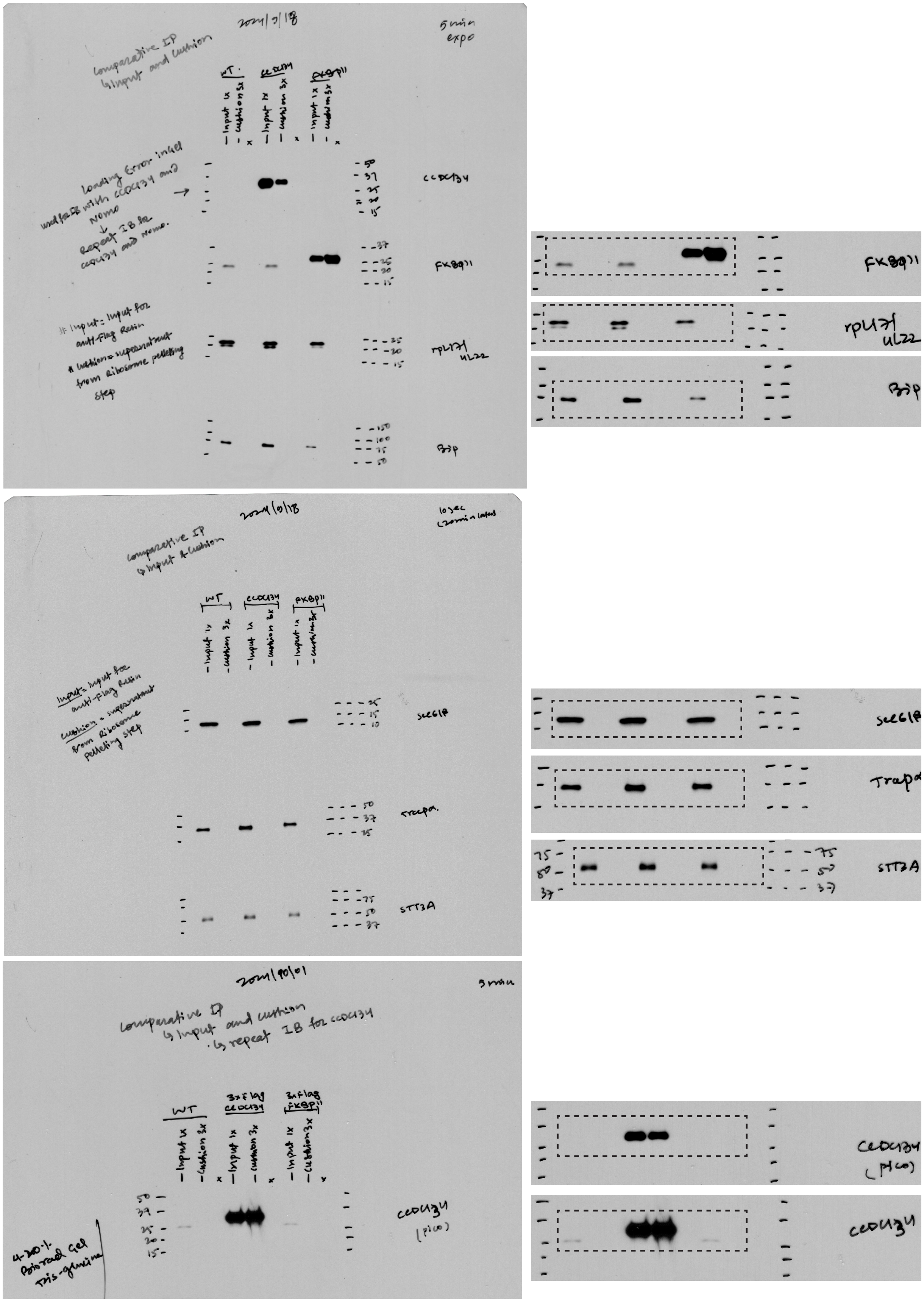

Extended Data Fig. 7a

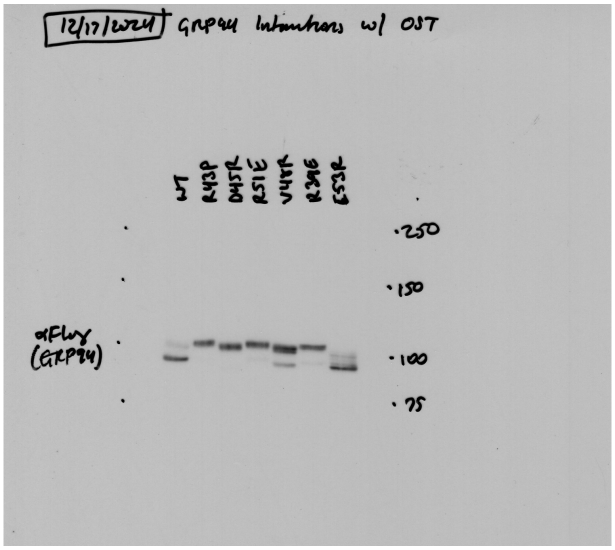

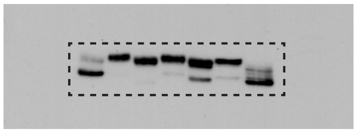

GRP94
(anti-Flag)
(longer exposure)

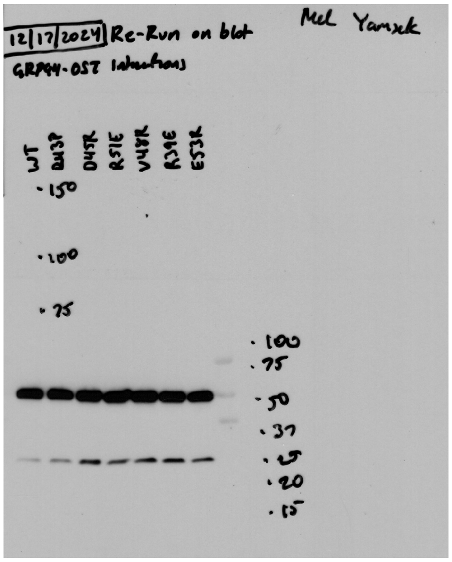

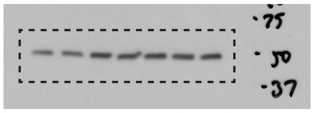

α-tubulin
(shorter exposure)

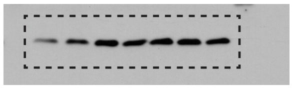

CCDC134
(longer exposure)

Extended Data Fig. 7b

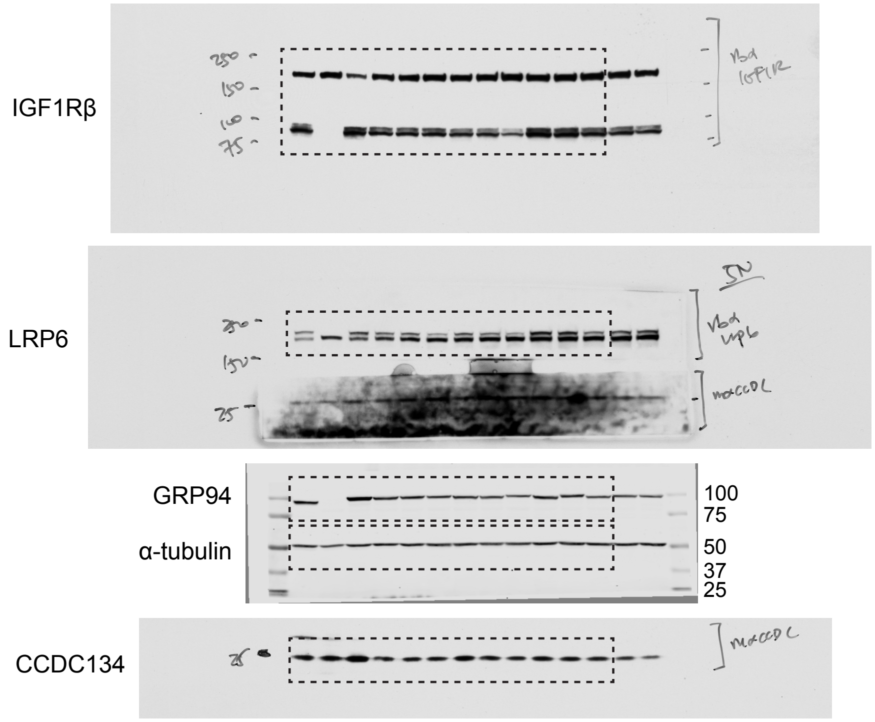

Extended Data Fig. 7c

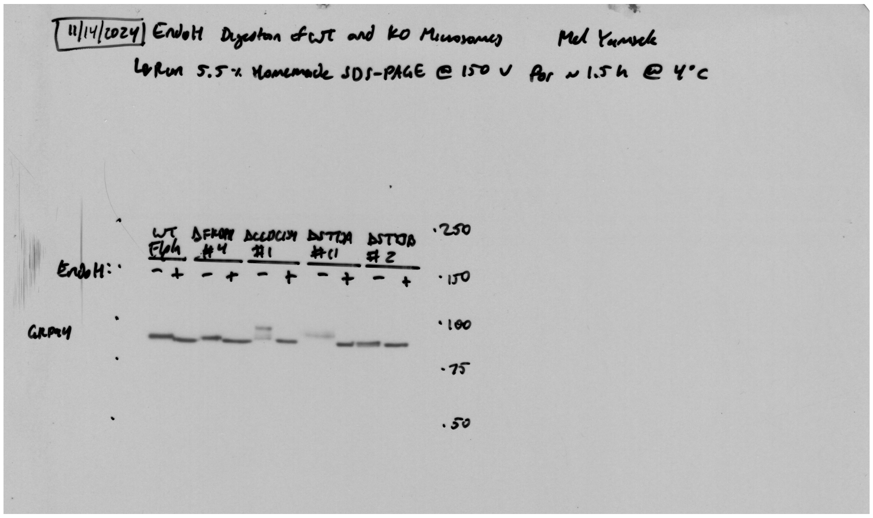

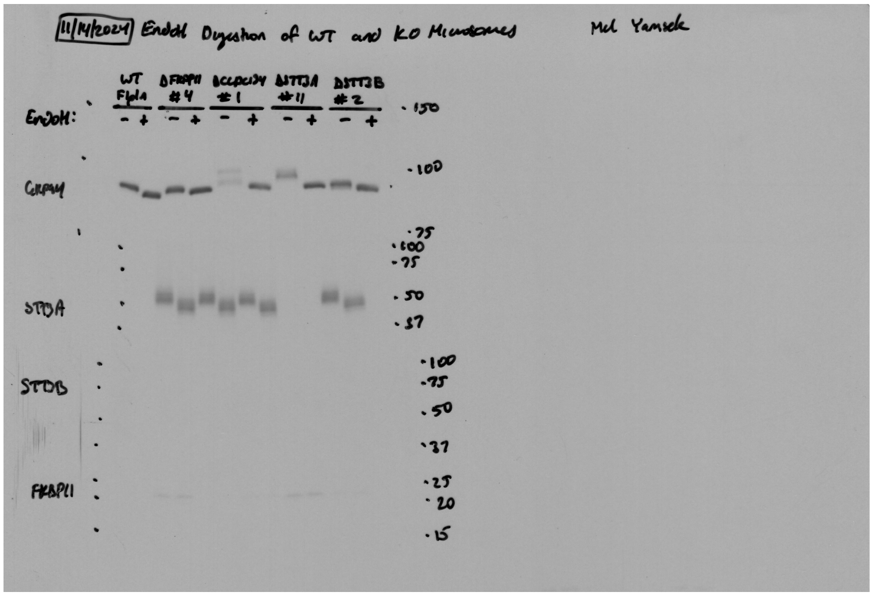

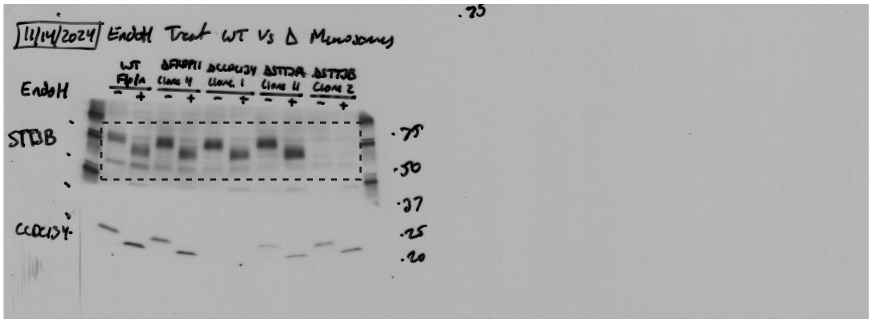

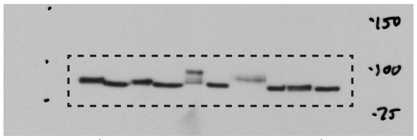

GRP94

(longer exposure)

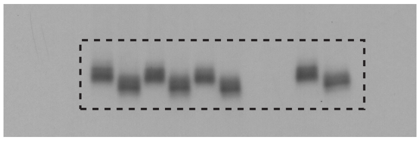

STT3A

(longer exposure)

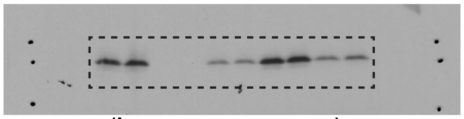

FKBP11

(longer exposure)

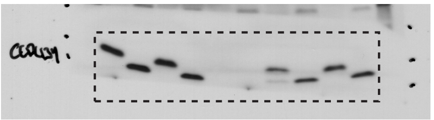

CCDC134

(longer exposure)

Extended Data Fig. 7d

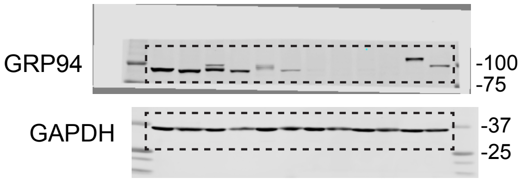

Extended Data Fig. 7e

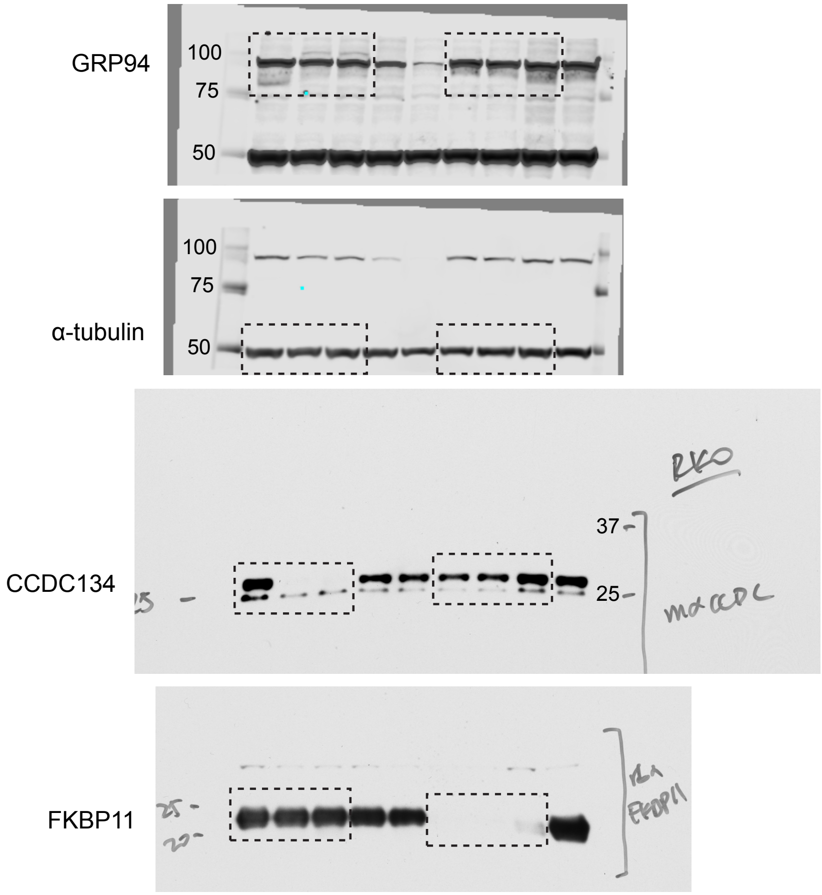

Extended Data Fig. 7f

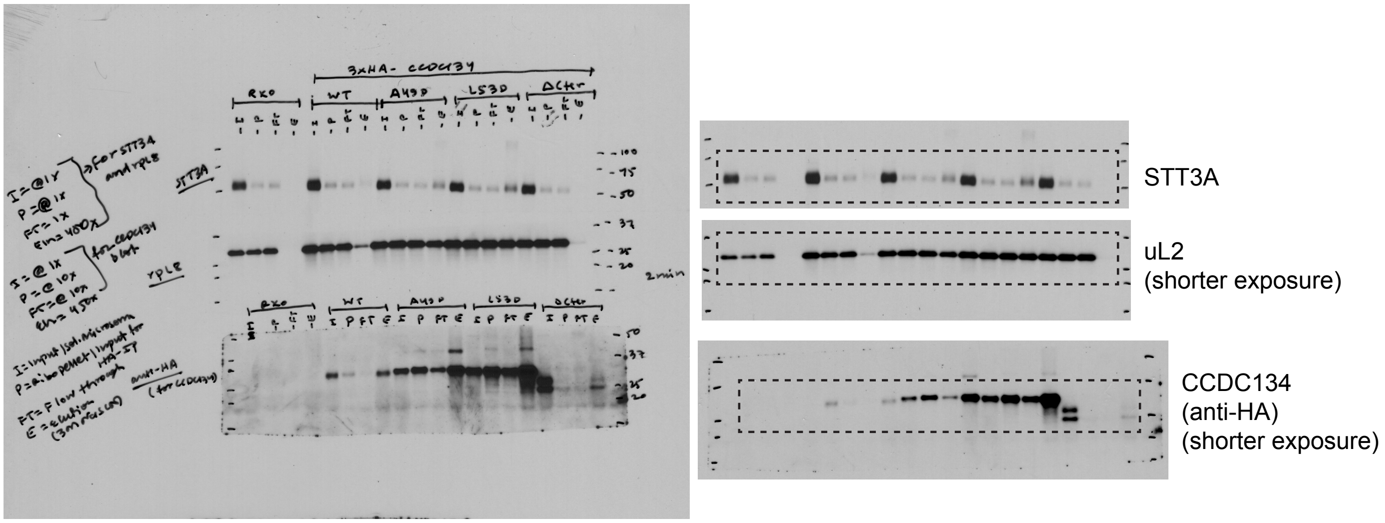

Extended Data Fig. 7g

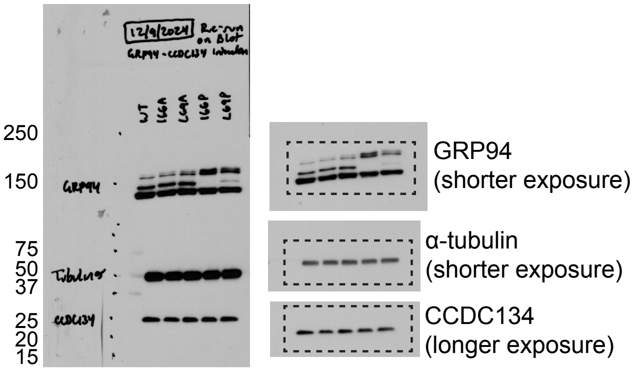

Supplement: Supplementary file 1 — Gel source data. [file 41586_2025_9756_MOESM1_ESM.pdf]
