# Supplementary material for: Structural basis of regulated N-glycosylation at the secretory translocon
Source: Nature. 2025 Nov 19;649(8097):777–84. doi: 10.1038/s41586-025-09756-8 (PMC12804085; doi:10.1038/s41586-025-09756-8)
Supplement: Supplementary file 2 — Reporting Summary [file 41586_2025_9756_MOESM2_ESM.pdf]

nature portfolio

Corresponding author(s): Robert J. Keenan
Rajat Rohatgi

Last updated by author(s): 10/03/2025

# Reporting Summary

Nature Portfolio wishes to improve the reproducibility of the work that we publish. This form provides structure for consistency and transparency in reporting. For further information on Nature Portfolio policies, see our Editorial Policies and the Editorial Policy Checklist.

## Statistics

For all statistical analyses, confirm that the following items are present in the figure legend, table legend, main text, or Methods section.

| n/a | Confirmed | |
|---|---|---|
| ☐ | ☒ | The exact sample size (*n*) for each experimental group/condition, given as a discrete number and unit of measurement |
| ☐ | ☒ | A statement on whether measurements were taken from distinct samples or whether the same sample was measured repeatedly |
| ☐ | ☒ | The statistical test(s) used AND whether they are one- or two-sided<br>*Only common tests should be described solely by name; describe more complex techniques in the Methods section.* |
| ☒ | ☐ | A description of all covariates tested |
| ☐ | ☒ | A description of any assumptions or corrections, such as tests of normality and adjustment for multiple comparisons |
| ☐ | ☒ | A full description of the statistical parameters including central tendency (e.g. means) or other basic estimates (e.g. regression coefficient) AND variation (e.g. standard deviation) or associated estimates of uncertainty (e.g. confidence intervals) |
| ☐ | ☒ | For null hypothesis testing, the test statistic (e.g. *F*, *t*, *r*) with confidence intervals, effect sizes, degrees of freedom and *P* value noted<br>*Give P values as exact values whenever suitable.* |
| ☒ | ☐ | For Bayesian analysis, information on the choice of priors and Markov chain Monte Carlo settings |
| ☒ | ☐ | For hierarchical and complex designs, identification of the appropriate level for tests and full reporting of outcomes |
| ☐ | ☒ | Estimates of effect sizes (e.g. Cohen's *d*, Pearson's *r*), indicating how they were calculated |

*Our web collection on statistics for biologists contains articles on many of the points above.*

## Software and code

Policy information about availability of computer code

| | |
|---|---|
| Data collection | The following software was used for cryo-EM data collection and processing: EPU-3.5.1, RELION-5.0, MotionCor2, CTFFIND4.1, Topaz-0.2.5, DeepEMhancer-0.17 |
| Data analysis | The following software was used for model building, refinement and validation: Colabfold2 implementation of Alphafold2-multimer v3, COOT release 0.9.8.8, Phenix release 1.21.2-5419, and ChimeraX release 1.8. The following software was used to analyze the Rfoot-seq data: Bowtie v2.2.6; TopHat v2.1.0; HTSeq-count v2.0.3; python 3.10; samtools v1.9; deeptools v3.1.1; DeepTMHMM; RibORF 2.0. |

For manuscripts utilizing custom algorithms or software that are central to the research but not yet described in published literature, software must be made available to editors and reviewers. We strongly encourage code deposition in a community repository (e.g. GitHub). See the Nature Portfolio guidelines for submitting code & software for further information.

## Data

Policy information about availability of data

All manuscripts must include a data availability statement. This statement should provide the following information, where applicable:

- Accession codes, unique identifiers, or web links for publicly available datasets
- A description of any restrictions on data availability
- For clinical datasets or third party data, please ensure that the statement adheres to our policy

Sequencing data are available at the NCBI Gene Expression Omnibus (GEO) repository with accession number GSE303507. Protein models and cryo-EM maps are

available at the RCSB Protein Data Bank (PDB ID 9N9J, 9YGY) and the Electron Microscopy Data Bank (EMD-49171, EMD-72945), respectively. All other data are available in the main text or the supplementary materials.

## Research involving human participants, their data, or biological material

Policy information about studies with human participants or human data. See also policy information about sex, gender (identity/presentation), and sexual orientation and race, ethnicity and racism.

| | |
|---|---|
| Reporting on sex and gender | N/A |
| Reporting on race, ethnicity, or other socially relevant groupings | N/A |
| Population characteristics | N/A |
| Recruitment | N/A |
| Ethics oversight | N/A |

Note that full information on the approval of the study protocol must also be provided in the manuscript.

# Field-specific reporting

Please select the one below that is the best fit for your research. If you are not sure, read the appropriate sections before making your selection.

☒ Life sciences ☐ Behavioural & social sciences ☐ Ecological, evolutionary & environmental sciences

For a reference copy of the document with all sections, see nature.com/documents/nr-reporting-summary-flat.pdf

# Life sciences study design

All studies must disclose on these points even when the disclosure is negative.

| | |
|---|---|
| Sample size | No sample size calculations were performed. To verify reproducibility, all cell-based experiments were repeated in part or in full, on independent days and with different batches of cell. Sample sizes were sufficient to yield reproducible and consistent results. The cryo-EM structure was determined from a single sample (with 55,750 particles contributing to the final map). |
| Data exclusions | No data were excluded from the analysis. |
| Replication | The Rfoot-seq experiment shown in Fig 1b-f was performed in two biological replicates for Flag-FKBP11 and Flag-CCDC134. Experiments in Fig. 3e,h, Fig. 4e,f, Ext. Data Fig. 1b, and Ext. Data Fig. 7a,b,d,e,f,g were repeated in at least two independent experiments with similar results (except for the GRP94 E53R mutant in Ext. Data Fig. 7a, which was tested once). The experiment in Ext. Data Fig. 7c was repeated twice from whole cell lysate and once from microsomes, with similar results. |
| Randomization | Randomization was not done for functional assays because there is nothing to randomize. |
| Blinding | Blinding is not relevant or practical for the functional and structural work. |

# Reporting for specific materials, systems and methods

We require information from authors about some types of materials, experimental systems and methods used in many studies. Here, indicate whether each material, system or method listed is relevant to your study. If you are not sure if a list item applies to your research, read the appropriate section before selecting a response.

## Materials & experimental systems

| n/a | Involved in the study | |
|---|---|---|
| ☐ | ☒ | Antibodies |
| ☐ | ☒ | Eukaryotic cell lines |
| ☒ | ☐ | Palaeontology and archaeology |
| ☒ | ☐ | Animals and other organisms |
| ☒ | ☐ | Clinical data |
| ☒ | ☐ | Dual use research of concern |
| ☒ | ☐ | Plants |

## Methods

| n/a | Involved in the study | |
|---|---|---|
| ☒ | ☐ | ChIP-seq |
| ☒ | ☐ | Flow cytometry |
| ☒ | ☐ | MRI-based neuroimaging |

# Antibodies

| | |
|---|---|
| Antibodies used | The following primary antibodies were used: mouse anti-CCDC134 (Santa Cruz Biotechnology, #sc-393390, RRID:AB_3662100, 1:500); mouse anti-GRP94 (R&D Systems, #MAB7606, RRID:AB_3644153, 1:2000); mouse anti-GRP94 (Santa Cruz Biotechnology, #sc-393402, RRID:AB_2892568, 1:2000); rabbit anti-LRP6 (Cell Signaling Technology, #2560, RRID:AB_2139329, 1:1000); mouse anti-α-Tubulin (MilliporeSigma, #T6199, RRID:AB_477583, 1:10000); mouse anti-α-Tubulin (Abcam, #ab11304, RRID:AB_297909, 1:1000); mouse anti-STT3A (Abnova, #H00003703-M02, RRID:AB_530104, 1:1000); rabbit anti-STT3A (Proteintech, #12034-1-AP, RRID:AB_2877818, 1:1000); rabbit anti-IGF1Rβ (Cell Signaling Technology, #9750, RRID:AB_10950969, 1:1000); mouse anti-GAPDH (Proteintech, #60004-1-Ig, RRID:AB_2107436, 1:10,000); rabbit anti-PSAP (GeneTex, #GTX101064, RRID:AB_2037779, 1:1000); mouse anti-HA (GenScript, #A01244, RRID:AB_1289306, 1:1000); rabbit anti-HA (Bethyl, #A191-102, RRID:AB_2891412, 1:2000); rabbit anti-uL22 (Abcepta, #AP9892b, RRID:AB_10613776, 1:1000); rabbit anti-uL2 (Abcam, #ab169538, RRID:AB_2714187, 1:1000); rabbit anti-Sec61β (Cell Signaling Technology, #14648, RRID:AB_2798555, 1:1000); rabbit anti-TRAPα (Millipore Sigma, #HPA011276, RRID:AB_1857503, 1:1000); rabbit anti-STT3B (Proteintech, #15323-1-AP, RRID:AB_2198046, 1:1000); rabbit anti-FKBP11 (Atlas Antibodies, #HPA041709, RRID:AB_10794487, 1:1000); mouse anti-FLAG (Millipore Sigma, #F1804, RRID:AB_262044, 1:1000); mouse anti-BiP (BD Transduction Lab, #610978, RRID:AB_398291, 1:1000).<br><br>The following secondary antibodies conjugated to horseradish peroxidase were used: Peroxidase AffiniPure Donkey Anti-Mouse IgG (H+L) (Jackson ImmunoResearch Laboratories, #715-035-150, RRID: AB_2340770, 1:10,000); Peroxidase AffiniPure Donkey Anti-Rabbit IgG (H+L) (Jackson ImmunoResearch Laboratories, #111-035-144, RRID: AB_2307391, 1:10,000); Peroxidase Donkey Anti-Rabbit IgG (Fc specific) (Sigma Aldrich, #SAB3700863, RRID:AB_3675584, 1:10000); Peroxidase Rabbit Anti-Mouse IgG H&L (Abcam, #ab6728, RRID:AB_955440, 1:10000). Secondary antibodies conjugated to IRDye® 800CW were obtained from LI-COR (IRDye® 800CW Donkey anti-Mouse IgG Secondary Antibody, 1:10,000). |
| Validation | All commercial antibodies were validated by the manufacturers for specificity against human antigen. The following antibodies were additionally validated by knockout or mutant expression experiments in this manuscript: anti-CCDC134, anti-GRP94, anti-LRP6, anti-PSAP, anti-IGF1Rbeta, anti-FKBP11, and anti-STT3A. The following have been further validated in the indicated citations: anti-CCDC134 (PMID: 39509507, 32181939), anti-PSAP (PMID: 39509507, 27383987), anti-STT3A (Proteintech) (PMID: 39509507), anti-STT3A (Abnova) (PMID:36261522), anti-LRP6 (PMID: 39509507), anti-IGF1Rbeta (PMID: 39509507), anti-GRP94 (Santa Cruz Biotechnology) (PMID: 39509507), anti-FKBP11 (PMID: 39259761) |

# Eukaryotic cell lines

Policy information about cell lines and Sex and Gender in Research

| | |
|---|---|
| Cell line source(s) | RKO cells were purchased from ATCC, and Flp-In T-REx HEK293 Cell Line are from Invitrogen |
| Authentication | Parental RKO cell line came with a certificate of authentication from the vendor (ATCC) and was used without further validation. Flp-In T-REx 293 cell line was authenticated by the antibiotic resistance markers within its genome. All knockouts were validated by PCR amplification of the genomic locus and immunoblotting for the absence of protein. |
| Mycoplasma contamination | Cells were checked approximately every six months for mycoplasma contamination using the Universal Mycoplasma Detection kit (ATCC), and were found to be negative. |
| Commonly misidentified lines<br>(See ICLAC register) | None used. |

# Plants

| | |
|---|---|
| Seed stocks | N/A |
| Novel plant genotypes | N/A |
| Authentication | N/A |
